# The Clinical Role of Systemic Inflammation-Based Biomarkers in Predicting Mortality in Post-Pneumonectomy Bronchopleural Fistula: A Multicenter Retrospective Analysis

**DOI:** 10.3390/diagnostics15222902

**Published:** 2025-11-16

**Authors:** Omer Topaloglu, Kubra Nur Kilic, Sami Karapolat, Elvan Senturk Topaloglu, Atila Turkyilmaz, Buket Kaytaz Alkas, Aziz Gumus, Hasan Turut, Celal Tekinbas

**Affiliations:** 1 Department of Thoracic Surgery, Faculty of Medicine, Recep Tayyip Erdogan University, Rize 53100, Turkey; hasan.turut@erdogan.edu.tr; 2 Department of Thoracic Surgery, Faculty of Medicine, Karadeniz Technical University, Trabzon 61080, Turkey; kubranurseyis@yahoo.com (K.N.K.); samikarapolat@yahoo.com (S.K.); atilaturkyilmaz@hotmail.com (A.T.); buketkaytaz@gmail.com (B.K.A.); celaltekinbas3@hotmail.com (C.T.); 3 Department of Pulmonology, Faculty of Medicine, Recep Tayyip Erdogan University, Rize 53100, Turkey; elvan.senturktopaloglu@erdogan.edu.tr (E.S.T.); aziz.gumus@erdogan.edu.tr (A.G.)

**Keywords:** bronchopleural fistula, pneumonectomy, inflammation, NLR, CAR, SIRI, mortality, prognosis

## Abstract

**Background:** Post-pneumonectomy bronchopleural fistula (PPBPF), although infrequent, represents one of the most devastating complications after pneumonectomy, carrying high morbidity and mortality. Accurate risk stratification is essential for timely management. Systemic inflammation-based hematologic indices—such as neutrophil-to-lymphocyte ratio (NLR), C-reactive protein/albumin ratio (CAR), systemic inflammation response index (SIRI), systemic immune-inflammation index (SIII), prognostic immune-inflammation index (PIII), and platelet-to-lymphocyte ratio (PLR)—serve as accessible, low-cost biomarkers reflecting host immune status and inflammatory burden. This study aimed to evaluate their association with mortality risk in patients with PPBPF. **Methods:** A multicenter retrospective cohort of 33 PPBPF patients (2014–2023) was analyzed. Demographic, clinical, and laboratory data at diagnosis were retrieved. Inflammatory indices were calculated from hematologic parameters. Associations with mortality were assessed using receiver operating characteristic (ROC) curves and univariate logistic regression. Post hoc power analyses were performed for key biomarkers. **Results:** Nine patients (27.3%) died during follow-up. Non-survivors had significantly higher levels of all biomarkers (*p* < 0.05). ROC analysis identified NLR as the most powerful discriminatory marker (AUC: 0.862), while SIII, SIRI, and CAR also demonstrated high accuracy (AUC > 0.83). Optimal thresholds of NLR ≥ 12 and CAR ≥ 10 yielded 88.9% sensitivity, >80% specificity, and excellent negative predictive values (NLR: 94.4%; CAR: 94.7%). Post hoc power analysis demonstrated robust statistical power for SIRI (94.9%), CAR (87.2%), and SIII (84.5%). **Conclusions:** Systemic inflammation-based biomarkers, particularly NLR and CAR, show strong associations with mortality in PPBPF. Incorporating these indices into clinical practice may help identify patients at increased risk and facilitate tailored surveillance and management strategies.

## 1. Introduction

Pneumonectomy is a major surgical procedure for treating malignant and benign pulmonary diseases, but it carries substantial risks of morbidity and mortality [1]. One of the most serious complications after this surgery is post-pneumonectomy bronchopleural fistula (PPBPF), which is characterized by a pathological communication between the pleural space and airways. The reported incidence of PPBPF ranges from 1.5% to 12.5% [2,3,4,5]. PPBPF can lead to complications, such as empyema, sepsis, and multiorgan failure, increasing mortality rates to 16–72% [5,6,7,8].

Patient- and procedure-related factors implicated in the development of PPBPF include advanced age, malnutrition, neoadjuvant therapy, right pneumonectomy, residual tumor at the bronchial stump, postoperative infection, and prolonged mechanical ventilation [5,8,9,10]. Although prolonged antibiotic therapy, pleural drainage, and, in some cases, surgical repair are used in clinical management, there is currently no standardized treatment algorithm [6,11,12,13].

In recent years, hematologic biomarkers that reflect systemic inflammation and nutritional status—including neutrophil/lymphocyte ratio (NLR), platelet/lymphocyte ratio (PLR), systemic inflammation response index (SIRI), systemic immune-inflammation index (SIII), pan-immune-inflammation index (PIII), C-reactive protein/albumin ratio (CAR), and prognostic nutritional index (PNI)—have been shown to have prognostic value in various malignancies and postoperative complications [14,15,16,17,18,19]. These indices provide a holistic reflection of inflammatory burden, immune competence, and nutritional status and offer practical, low-cost, and easily obtainable tools for clinical assessment of disease severity and outcomes [20,21]. However, the roles of these biomarkers in characterizing mortality risk among PPBPF cases have not been adequately elucidated in the literature.

The aim of this study was to evaluate the value of systemic inflammation-based biomarkers (CAR, NLR, SIRI, SIII, PIII) in assessing mortality risk in patients with PPBPF. Accordingly, our hypothesis was that increases, particularly in CAR, SIRI, and SIII levels, would be strongly associated with higher mortality and poorer clinical prognosis. Establishing this relationship with receiver operating characteristic (ROC) analysis and post hoc power calculations would fill an important gap in the literature.

## 2. Methods

### 2.1. Study Design and Patient Selection

In this retrospective, multicenter observational study, a total of 33 consecutive adult patients who developed post-pneumonectomy bronchopleural fistula (PPBPF) at two different centers between January 2014 and January 2023 were evaluated. Patients were retrospectively reviewed using the hospital information management system and patient files. PPBPF diagnosis was established based on bronchoscopic evaluation findings in addition to clinical and radiological data. Patients whose medical records did not contain clear evidence supporting the diagnosis were excluded from the study.

The inclusion criteria were defined as follows: (1) having undergone pneumonectomy between 2014 and 2023, (2) having received PPBPF diagnosis during the postoperative follow-up period, and (3) having complete access to complete blood count and biochemical laboratory data at the time of diagnosis.

To ensure the homogeneity of the study group and evaluate the prognostic effects of systemic inflammatory markers more reliably, the following patients were excluded: those with missing clinical or laboratory data, who had undergone thoracic surgery on the contralateral hemithorax before or after pneumonectomy, who underwent extended pneumonectomy, those with a history of previous or synchronous other primary malignancy, and who had received neoadjuvant chemotherapy or radiotherapy. Additionally, PPBPF cases occurring within the first postoperative week (i.e., postoperative days 1–7) were excluded. Early PPBPF is typically attributed to mechanical or technical factors—such as inadequate bronchial stump closure or stump ischemia—rather than systemic inflammatory response. As these early fistulas are closely associated with surgical technique rather than inflammatory biology, their exclusion enabled a more accurate assessment of the prognostic value of inflammation-based biomarkers by reducing confounding from non-inflammatory etiologies [22].

### 2.2. Collection of Clinical and Laboratory Data

Laboratory values from the preoperative and early postoperative periods for all patients were obtained from the hospital information system. For complete blood count, an adequate volume of blood was collected into ethylenediaminetetraacetic acid (EDTA)-containing vacuum tubes. Hematological analyses were performed using a Beckman Coulter UniCel^®^ DxH 800 analyzer (Beckman Coulter Inc., Brea, CA, USA).

All laboratory parameters used to calculate inflammatory indices were obtained at the time of PPBPF diagnosis, coinciding with bronchoscopic confirmation of the fistula. Serial or longitudinal measurements were not available due to the retrospective design.

Analyzed laboratory parameters:

- CRP (C-reactive protein): mg/L
- Albumin: g/dL
- Neutrophil, lymphocyte, eosinophil, monocyte, and platelet counts: ×10^3^/mm^3^ (cells/μL)

### 2.3. Calculation of Indices and Scores

Various hematologic indices described in the literature were used to evaluate the systemic inflammatory response of the patients [23]. All formulas are presented below:

- Neutrophil-to-Lymphocyte Ratio (NLR) = Neutrophil/Lymphocyte
- Eosinophil-to-Lymphocyte Ratio (ELR) = Eosinophil/Lymphocyte
- Platelet-to-Lymphocyte Ratio (PLR) = Platelet/Lymphocyte
- CRP/Albumin Ratio (CAR) = CRP/Albumin
- Prognostic Nutritional Index (PNI) = [10 × Albumin] + [0.005 × Lymphocyte (cells/mm^3^)]
- Systemic Inflammation Response Index (SIRI) = (Neutrophil × Monocyte)/Lymphocyte
- Systemic Immune-Inflammation Index (SIII) = (Neutrophil × Platelet)/Lymphocyte
- Pan-Immune-Inflammation Index (PIII/PIV) = (Neutrophil × Platelet × Monocyte)/Lymphocyte

All values were standardized using neutrophil, lymphocyte, monocyte, eosinophil, and platelet count expressed as ×10^3^/mm^3^, CRP in mg/L, and albumin in g/dL.

### 2.4. Statistical Analysis

Descriptive statistics were presented as mean ± standard deviation, median (interquartile range: IQR), and percentage (%). The normality of continuous variables was assessed with the Kolmogorov–Smirnov test; comparisons were made using Student’s t-test or the Mann–Whitney U test as appropriate. Categorical variables were analyzed using the chi-square or Fisher’s exact tests. Univariable logistic regression analysis was applied to variables associated with mortality.

Receiver operating characteristic (ROC) curve analysis was performed to evaluate the prognostic performance of systemic inflammatory markers, and sensitivity, specificity, and positive and negative predictive values were calculated. A two-sided *p*-value < 0.05 was considered statistically significant. All analyses were conducted using IBM SPSS Statistics for Windows, version 26.0 (IBM Corp., Armonk, NY, USA).

Given the extremely rare nature of PPBPF, a conventional a priori sample size calculation was not feasible. This study, therefore, adopts an exploratory, hypothesis-generating design in this high-mortality, low-incidence population. Because the sample size for this retrospective study was not predetermined, a post hoc power analysis based on the obtained data was performed. Analyses focused on the systemic inflammatory markers most strongly associated with mortality: SIRI, CAR, and SIII. Statistical power was 94.9% for SIRI, 87.2% for CAR, and 84.5% for SIII. Power values for the other markers, NLR, PIII, and PLR, ranged between 74% and 78%, indicating that interpretation of the findings for these parameters should be undertaken with greater caution. Despite the modest cohort size, these power estimates support the statistical adequacy and robustness of the primary findings in the context of this rare disease.

## 3. Results

**Figure 1:**
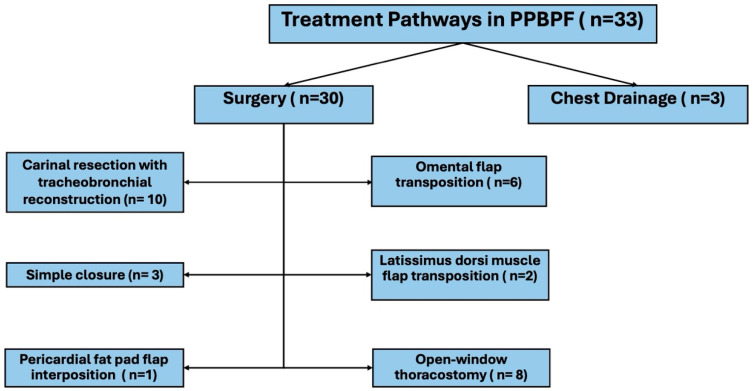
Treatment pathways in patients with post-pneumonectomy bronchopleural fistula (PPBPF).

A total of 33 patients who developed PPBPF were included in the study. The mean age of the patients was 59 ± 12 years, and the vast majority were male (*n* = 32, 97%). Of the cases, 81.8% (*n* = 27) had a diagnosis of bronchial carcinoma, whereas 18.2% (*n* = 6) had a benign etiology. Right pneumonectomy was performed in 26 patients (78.8%), and left pneumonectomy in seven patients (21.2%). Surgical fistula repair was performed in only 30 of these patients (90.9%); the remaining cases were managed with tube thoracostomy. The distribution of treatment modalities applied in PPBPF patients is illustrated in Figure 1. During follow-up, nine patients (27.3%) died.

The comparison between survivors and non-survivors is summarized in Table 1. There were no significant differences between the groups with respect to basic variables, such as age and sex; however, statistically significant differences were observed for follow-up duration (*p* = 0.018) and length of intensive care unit (ICU) stay (*p* = 0.017).

Laboratory findings showed that serum CRP levels (*p* = 0.003) and all systemic inflammatory markers were significantly higher in non-survivors. Significant differences were observed, particularly in SIRI (*p* < 0.001), NLR (*p* = 0.001), SIII (*p* = 0.002), and PIII (*p* = 0.004) values.

**Figure 2:**
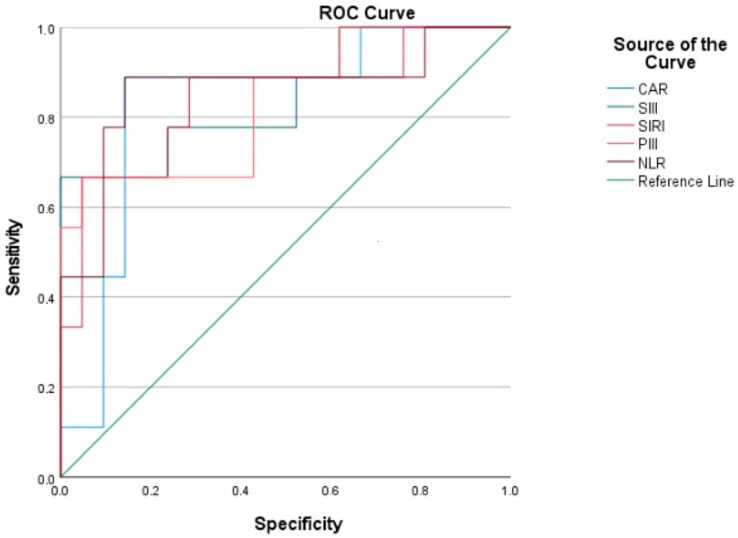
ROC curves showing the prognostic performance of systemic inflammation-based biomarkers for mortality in PPBPF patients, with NLR demonstrating the highest AUC.

All evaluated systemic inflammatory markers (CAR, NLR, SIRI, SIII, and PIII) demonstrated a statistically significant association with mortality (all *p* < 0.05). Univariable logistic regression analysis identified all of these markers as significant risk indicators associated with mortality (*p* < 0.05; Table 2). According to ROC analysis results, the highest AUC was observed for NLR (AUC: 0.862; *p* = 0.002), followed by SIII, SIRI, CAR, and PIII (AUC > 0.83; *p* < 0.01) (Table 3; Figure 2).

Further analyses using cut-off values, thresholds of CAR ≥ 10 and NLR ≥ 12 yielded a sensitivity of 88.9% and specificity of >80% (Table 4). To improve interpretability and support clinical translation of these findings, a concise summary of the ROC-derived cut-off values and their potential clinical relevance is provided in Table 5.

## 4. Discussion

This study evaluated the role of systemic inflammation-based biomarkers in assessing mortality among patients who developed PPBPF and revealed six principal findings:

(a) Levels of SIRI, CAR, SIII, NLR, PIII, and PLR were significantly higher in cases with fatal outcomes (all *p* < 0.05).
(b) Each marker was independently associated with mortality in univariable logistic regression analyses.
(c) ROC analysis identified NLR as having the highest AUC, along with SIII, SIRI, CAR, and PIII, also demonstrating high discriminative performance.
(d) Thresholds of CAR ≥ 10 and NLR ≥ 12 provided strong clinical discrimination with 88.9% sensitivity, specificity of >80%, and high negative predictive values (NPV).
(e) Post hoc power analyses for SIRI, CAR, and SIII indicated high statistical power (84.5–94.9%).
(f) CRP, CAR, and SIRI levels as well as ICU length of stay were significantly higher in fatal cases.

CAR is an effective biomarker for assessing tumor progression and postoperative complications in advanced-stage lung cancer and other solid tumors [24,25]. Similarly, SIRI has notable prognostic value for survival in gastrointestinal and respiratory system malignancies [26,27]. SIII, which integrates neutrophil, lymphocyte, and platelet parameters, is regarded as a holistic indicator of systemic inflammation, and elevated levels have been associated with poor prognosis in various malignancies, including non-small cell lung cancer (NSCLC) [28,29]. In the present study, the significant elevation of these markers in fatal cases indicates that inflammation-based biomarkers reflect tumor biology as well as the systemic inflammatory response associated with postoperative complications, as supported by statistically higher levels observed in non-survivors (all *p* < 0.05). Although only the values at the time of PPBPF diagnosis were examined, dynamic monitoring of these parameters in the pre- and postoperative period may improve complication-risk assessment and enable more individualized patient management.

Unlike malignant conditions, where inflammation-based biomarkers primarily reflect tumor burden and chronic immune dysregulation, PPBPF represents an acute, catastrophic postoperative complication characterized by bronchopleural contamination, septic systemic inflammatory response, and rapid physiologic deterioration. Therefore, elevated biomarker levels in PPBPF likely reflect the severity of septic-inflammatory activation rather than tumor biology. This distinction highlights PPBPF as a unique clinical entity in which inflammatory indices serve as markers of acute inflammatory burden and short-term mortality risk, underscoring their potential role in early risk stratification and proactive clinical management.

The finding that all markers were significantly associated with mortality in univariable logistic regression suggests that each may serve as a potential standalone risk indicator. Similar results have been reported in the literature. Zhou et al. reported that CAR is associated with survival as well as ICU admission and complication risk in lung cancer [30]. Qi et al. reported that SIRI has independent prognostic value in advanced-stage lung cancer [31]. Yehong Liu et al. demonstrated that SIII is a strong marker for the development and severity of coronary artery disease [32]. The data from our study are consistent with these reports and, by focusing specifically on patients with PPBPF, provide additional, disease-specific evidence with potential clinical applicability.

NLR has prognostic value in many solid tumors as a robust indicator of tumor progression and systemic inflammatory response [16,33]. In the present study, AUC values of >0.83 for SIII, SIRI, CAR, and PIII demonstrated the high discriminatory power of these markers for PPBPF-related mortality. The optimal thresholds identified in ROC analysis (NLR > 4.93; SIII > 1.069,9; SIRI > 2.05; CAR > 0.29; PIII > 4.73) support that PPBPF is not merely a local complication but one shaped by systemic inflammatory response. Particularly, easily measurable parameters like NLR and SIRI emerge as simple, low-cost tools with high clinical impact potential for mortality risk assessment when used individually or in combination.

NLR has been established as a prognostic marker in numerous clinical conditions ranging from malignancies to cardiovascular diseases [34]. Elevated neutrophils are linked to inflammatory cytokine release and an immunosuppressive tumor microenvironment, whereas low lymphocytes reflect impaired anti-tumor immune response and poor prognosis [16]. Templeton et al. reported that high NLR values, particularly at a threshold of ≥5, correlate with increased mortality in solid tumors [35]. The NLR cut-off of ≥12 identified in the present study exceeds this literature threshold, offering a more specific risk definition for high-mortality complications like PPBPF. This threshold yielded 88.9% sensitivity, 80.9% specificity, 72.7% PPV, and 94.4% NPV. Mortality rates were 5.6% in patients with NLR < 12 versus significantly higher rates above this threshold. Integration of this threshold into clinical practice could facilitate early identification of high-risk patients, personalization of monitoring strategies, and timely planning of supportive therapies.

From a clinical standpoint, the NLR ≥ 12 and CAR ≥ 10 thresholds identified in this study should not be interpreted as absolute treatment triggers, but rather as early clinical alert signals for patients at risk of rapid deterioration after pneumonectomy. In patients exceeding these thresholds, closer hemodynamic monitoring, more frequent inflammatory laboratory assessment, and early escalation of supportive measures—including prompt sepsis evaluation, aggressive source control strategies, and consideration of timely ICU transfer when clinically indicated—may be warranted. By serving as practical bedside indicators of escalating systemic inflammatory burden, these biomarkers have the potential to facilitate proactive, individualized postoperative surveillance in PPBPF. To support clinical translation of these findings, a concise summary of the ROC-derived cut-off values and their potential clinical relevance is presented in Table 5.

CAR combines CRP and albumin levels to reflect both inflammation and nutritional reserve. Elevated CAR is associated with poor prognosis in multiple malignancies [24,25]. Lu et al. reported that CAR > 0.1 is inversely associated with survival in NSCLC [36]. Zhou et al. demonstrated a 1.34-fold increased mortality risk in SCLC patients with CAR > 0.441 [37]. The CAR cut-off of ≥10 obtained in the present study is notably higher than oncological thresholds reported in the literature, enabling more precise determination of PPBPF-specific mortality risk. This threshold achieved 88.9% sensitivity, 80.0% specificity, 72.7% PPV, and 94.7% NPV. Mortality rates were 5.3% for patients with CAR < 10 and 72.7% for those with CAR ≥ 10. The high PPV and NPV indicate that CAR is a robust clinical tool for both ruling out and indicating mortality risk.

SIII, which combines neutrophil, lymphocyte, and platelet counts, reflects systemic inflammation and is associated with poor prognosis in solid malignancies, such as lung, gastric, and colorectal cancer [28,29]. PIII is a novel composite score that simultaneously assesses tumor burden, immune response, and inflammatory status. In the present study, both parameters demonstrated strong discriminatory power for mortality with high AUC values. Integration of these parameters into clinical practice could make an important contribution to early risk stratification and targeted complication management, particularly in high-mortality entities such as PPBPF.

SIRI, comprising neutrophil, monocyte, and lymphocyte ratios, is a powerful prognostic marker, particularly in respiratory and gastrointestinal malignancies [26,27]. High SIRI levels are associated with immunosuppression, proinflammatory cytokine production, and increased cellular stress, enhancing susceptibility to both tumor progression and postoperative complications [20,33]. To our knowledge, no prior study has evaluated the prognostic value of SIRI specifically in a cohort of patients who developed PPBPF; in this respect, our analysis is the first to assess this index in context of this complication. The significantly higher SIRI, SIII, and PIII values observed in fatal cases in the present study indicate that these indices may serve as strong biomarkers for assessing PPBPF-related mortality and underscore the clinical relevance of integrated inflammation scores in prognostic assessment.

The strong prognostic effect of inflammatory markers demonstrates that inflammation is not only a biological response, but also a determinant of clinical outcomes [38,39]. CRP, as a classical marker reflecting the extent of tissue injury and systemic inflammation, is shown to be associated with mortality in numerous studies [40]. CAR, by assessing inflammation together with nutritional status, emerges as a sensitive marker that enables the early recognition of adverse clinical courses [25]. SIRI and infection-related inflammatory processes within the tumor microenvironment are associated with poor prognosis [31]. In the present study, increases in these markers were accompanied by prolonged ICU length of stay, with all differences reaching statistical significance (*p* < 0.05). This finding supports the notion that a high systemic inflammatory response delays postoperative recovery and increases the risk of complications. Conversely, the significantly shorter total follow-up time observed among fatal cases suggests that these patients died earlier and that inflammatory burden may directly affect survival time. The finding that CRP, CAR, and SIRI levels were significantly elevated in fatal cases—when interpreted alongside clinical parameters, such as ICU length of stay—indicates that inflammation is a biochemical phenomenon and a decisive factor shaping the clinical course. Therefore, it is evident that inflammatory markers and associated clinical variables (e.g., ICU length of stay) should be evaluated in a holistic manner for risk assessment. Such an approach could provide a holistic model that improves risk stratification accuracy in patients who develop PPBPF.

In light of these results, systemic inflammation-based biomarkers appear to be diagnostically and prognostically valuable in patients with BPF after pneumonectomy. Our study represents one of the first analyses focusing exclusively on patients who developed BPF; it provides cut-off values for parameters, such as CAR, SIRI, SIII, NLR, PIII, and PLR in this patient group; and offers a comparative assessment of the clinical performance of biomarkers using post hoc power analyses. Therefore, it makes a novel and clinically meaningful contribution to the literature. Moreover, by examining the relationships of different biomarkers at the biochemical level along with survival and clinical course in a multifaceted manner, this approach aims to fill an existing knowledge gap. In this respect, our study may guide complication risk stratification in clinical practice and inform the design of future, larger prospective investigations.

Given the rarity and high mortality of PPBPF, the sample size in this study reflects one of the largest contemporary cohorts yet remains insufficient to draw broad generalizations. Therefore, the ROC-based thresholds of NLR ≥ 12 and CAR ≥ 10 should be interpreted as hypothesis-generating findings rather than definitive clinical cut-offs. These elevated values likely reflect the intense systemic inflammatory response characteristic of PPBPF, which differs from oncology-specific thresholds frequently reported in the literature. Future studies with larger, multi-institutional datasets are required to externally validate these cut-offs and further refine their applicability in routine practice.

Because cause-specific mortality could not be classified in detail, the inflammatory biomarkers in this study should be interpreted as indicators associated with mortality rather than definitive causal predictors. These findings, therefore, remain hypothesis-generating and require prospective validation in cohorts with complete clinical endpoint documentation.

Although only univariable logistic regression was performed, this approach was deliberate to avoid model overfitting given the limited number of mortality events in this rare-event dataset. This conservative statistical strategy ensured stable estimates and preserved analytical validity. Nevertheless, exploratory multivariable analyses incorporating key clinical covariates will be essential in future prospective multicenter cohorts to confirm the independent prognostic contribution of these inflammatory biomarkers.

Despite these constraints, the strong effect sizes, high AUC values, and robust post hoc power levels (84.5–94.9% for key markers) support the reliability of the findings. However, the retrospective nature of the study, lack of standardized comorbidity indices (e.g., Charlson or Elixhauser indices), and absence of serial biomarker assessment underscore the need for larger prospective and comparative studies to further validate these results. Although post hoc power analysis demonstrated adequate power for key biomarkers, these results should still be regarded as exploratory, and prospective multicenter validation remains essential before clinical implementation of these inflammatory indices.

## 5. Limitations

This study has several limitations. First, its retrospective, multicenter design is susceptible to potential selection bias and data loss. Although inclusion and exclusion criteria were defined carefully, the inherent limitations of retrospective data collection cannot be completely eliminated. Second, the analysis focused exclusively on pneumonectomy patients who developed BPF; the absence of a control group limits the absolute interpretation of the discriminatory power of the biomarkers. Third, the cut-off values for systemic inflammatory markers were determined from the study sample, and their validity in broader and different populations has not yet been established. Fourth, all biomarkers were measured only at the time of PPBPF diagnosis by FOB; therefore, temporal dynamics and the effects of long-term monitoring could not be assessed. In particular, serial or longitudinal laboratory measurements were not available due to the retrospective design, limiting the ability to evaluate dynamic changes in systemic inflammatory response over time. Fifth, the relatively small sample size (*n* = 33) inherently limits statistical power and precludes the ability to perform multivariable regression analyses. Although PPBPF is a rare and highly morbid postoperative complication, larger, multi-institutional datasets are necessary to validate these findings and to allow robust multivariable adjustment. In addition, baseline comorbidity indices (e.g., Charlson Comorbidity Index or Elixhauser Index) were not available, which may have limited adjustment for pre-existing clinical risk factors. Furthermore, although all *p*-values were reported and statistical tests were applied appropriately, the modest cohort size limited the feasibility of applying multiple-comparison corrections (e.g., Bonferroni or Tukey adjustments).

Although post hoc power analysis demonstrated adequate power for key biomarkers, these results should still be regarded as exploratory rather than confirmatory, and prospective multicenter validation is required before clinical implementation of these inflammatory indices.

Mortality was evaluated as all-cause mortality, because detailed cause-specific classification was not consistently available; therefore, the observed relationships between biomarker levels and mortality should be interpreted as associations rather than definitive causal predictors.

For these reasons, our findings require validation in larger, prospective, multicenter and comparative studies.

## 6. Conclusions

The prognostic value of systemic inflammatory markers is increasingly recognized in patients who develop PPBPF. In this study, composite inflammation scores such as CAR, SIRI, and SIII demonstrated strong statistical associations with mortality, suggesting potential utility in early risk assessment. However, these findings should be interpreted as exploratory rather than confirmatory, given the limited sample size and lack of cause-specific mortality classification. The threshold values identified by ROC analyses should therefore be regarded as hypothesis-generating indicators rather than definitive clinical predictors. Our results suggest that development of novel biomarkers that integrate inflammatory response and nutritional status may guide earlier identification of patients at risk of adverse outcomes, although external validation in larger prospective cohorts is essential before clinical application. Accordingly, incorporation of systemic inflammation-based parameters into routine post-pneumonectomy monitoring algorithms—once validated—could improve the precision of risk stratification and contribute to individualized patient management.

## Figures and Tables

**Table 1:** Clinical Comparison of Survivors and Non-Survivors in Post-Pneumonectomy Bronchopleural Fistula (PPBPF).

Parameters	Survivors (*n* = 24)	Non-Survivors (*n* = 9)	*p*-Value
Age (Year)	57 ± 13	65 ± 7	0.066
Sex (Female/Male)	1/23	0/9	0.727
Follow-up period (days)	438 (241–1510)	51(15–411)	0.018
Patients undergoing fistula repair, *n* (%)	5 (21)	3 (33)	0.374
Pneumonectomy side-right, *n* (%)	20 (83)	6 (67)	0.297
Ward length of stay (days)	5 (3–7)	3 (2–7)	0.112
ICU length of stay (days)	2(2–2)	3 (2–11)	0.017
Total length of hospital stays (days)	7 (5–9)	7 (5–22)	0.742
Creatinine	0.71 (0.60–0.85)	0.70 (0.63–0.91)	0.722
Albumin	29.5 ± 5.5	26.6 ± 4.0	0.161
CRP	201 ± 94	323 ± 95	0.003
CAR	7.3 ± 4.2	12.2 ± 3.3	0.005
SIRI	7.9 ± 6.2	20.7 ± 11.4	<0.001
SIII	2959 (1194–4278)	6230 (3401–10,856)	0.002
PIII	2181 (818–4867)	8920 (2738–13,877)	0.004
NLR	7.3 (%4.5–11.3)	14.1 (%12.5–32.9)	0.001
PLR	284 ± 147	521 ± 222	0.002

**Abbreviations:** CRP = C-reactive protein; CAR = C-reactive protein-to-albumin ratio; SIRI = systemic inflammation response index; SIII = systemic immune-inflammation index; PIII = prognostic immune-inflammation index; NLR = neutrophil-to-lymphocyte ratio; PLR = platelet-to-lymphocyte ratio. Data are presented as mean ± standard deviation, median (IQR 25–75%), and *n* (%).

**Table 2:** Univariate binary logistic regression analysis of inflammatory markers Predicting Mortality.

Inflammatory Marker	OR	95% Confidence Interval	*p*-Value
CRP	1.016	1.003–1.030	0.019
CAR	1.374	1.052–1.793	0.020
SIRI	1.207	1.044–1.395	0.011
SIII	1.001	1.000–1.001	0.019
PIII	1.000	1.000–1.001	0.012
NLR	1.254	1.028–1.530	0.026
PLR	1.007	1.002–1.013	0.014

**Table 3:** ROC analysis demonstrating prognostic performance of systemic inflammatory markers for mortality.

Inflammatory Marker	Area	Std. Error	95% Confidence Interval	*p*-Value
CAR	0.831	0.084	0.666–0.996	0.005
SIII	0.847	0.086	0.678–1.000	0.003
SIRI	0.841	0.087	0.670–1.000	0.004
PIII	0.831	0.087	0.659–1.000	0.005
NLR	0.862	0.088	0.690–1.000	0.002

**Table 4:** Prognostic performance of systemic inflammatory markers for survival.

Cut-off Values	Sensitivity	Specificity	PPV	NPV
CAR ≥ 10	88.9%	85.7%	72.7%	94.7%
NLR ≥ 12	88.9%	80.9%	66.7%	94.4%
SIRI ≥ 10	88.9%	66.7%	53.3%	93.3%
SIII ≥ 4500	66.7%	76.2%	54.5%	84.2%
PIII ≥ 6000	66.7%	90.5%	75%	86.4%

**Table 5:** ROC-derived optimal cut-off values for systemic inflammatory biomarkers and their potential clinical interpretation in patients with post-pneumonectomy bronchopleural fistula (PPBPF).

Cut-off Values	Interpretation	Suggested Clinical Consideration
CAR ≥ 10	High inflammatory burden with impaired nutritional status	Evaluate the inflammatory and nutritional status closely
NLR ≥ 12	Marked systemic inflammation	Consider closer monitoring and early escalation of care if clinical deterioration occurs
SIRI ≥ 10	Immune dysregulation and heightened inflammatory activation	Maintain heightened awareness of sepsis risk and adverse outcomes
SIII ≥ 4500	Increased neutrophil–platelet activation with lymphocyte suppression	May assist in identifying patients at increased risk of poor outcome
PIII ≥ 6000	Combined inflammation–immunity index indicating systemic stress	Suggests need for careful postoperative surveillance and early supportive strategies

**Note:** This table is provided for descriptive clarity. Clinical decisions should not rely solely on these exploratory values.

## Data Availability

All data generated or analyzed during this study are included in this article. The data will be available upon reasonable request (contact persons: omer.topaloglu@erdogan.edu.tr) (the data are not publicly available due to ethical restrictions).
